# Transmissibility of MERS-CoV Infection in Closed Setting, Riyadh, Saudi Arabia, 2015

**DOI:** 10.3201/eid2510.190130

**Published:** 2019-10

**Authors:** Maria D. Van Kerkhove, Sadoof Alaswad, Abdullah Assiri, Ranawaka A.P.M. Perera, Malik Peiris, Hassan E. El Bushra, Abdulaziz A. BinSaeed

**Affiliations:** Institut Pasteur, Paris, France (M.D. Van Kerkhove);; Ministry of Health, Riyadh, Saudi Arabia (S. Alaswad, A. Assiri, H.E. El Bushra, A.A. BinSaeed);; Jubail General Hospital, Riyadh (S. Alaswad);; The University of Hong Kong, Hong Kong, China (R.A.P.M. Perera, M. Peiris);; HKU-Pasteur Research Pole, Hong Kong (M. Peiris)

**Keywords:** MERS-CoV, Middle East respiratory syndrome coronavirus, human-to-human transmission, seroepidemiology, outbreak investigation, viruses, Riyadh, Saudi Arabia

## Abstract

To investigate a cluster of Middle East respiratory syndrome (MERS) cases in a women-only dormitory in Riyadh, Saudi Arabia, in October 2015, we collected epidemiologic information, nasopharyngeal/oropharyngeal swab samples, and blood samples from 828 residents during November 2015 and December 2015–January 2016. We found confirmed infection for 19 (8 by reverse transcription PCR and 11 by serologic testing). Infection attack rates varied (2.7%–32.3%) by dormitory building. No deaths occurred. Independent risk factors for infection were direct contact with a confirmed case-patient and sharing a room with a confirmed case-patient; a protective factor was having an air conditioner in the bedroom. For 9 women from whom a second serum sample was collected, antibodies remained detectable at titers >1:20 by pseudoparticle neutralization tests (n = 8) and 90% plaque-reduction neutralization tests (n = 2). In closed high-contact settings, MERS coronavirus was highly infectious and pathogenicity was relatively low.

Middle East respiratory syndrome (MERS) coronavirus (CoV) is a zoonotic virus (*1*). Approximately 2,266 laboratory-confirmed cases of MERS have been reported to the World Health Organization (WHO) (*2*) since the identification of the first human cases in 2012 (*3*,*4*).

Although the primary source of human infections is MERS-CoV–infected dromedaries, the modes of transmission from dromedaries to humans remain unclear (*5*). Human-to-human transmission has occurred primarily in healthcare settings (*6*), sometimes resulting in large explosive outbreaks (*7*,*8*). However, to date, no sustained human-to-human infection has been detected. Few outbreaks of MERS-CoV outside of healthcare settings have been documented, and limited transmission within families has been reported, but secondary attack rates in households or in settings outside of healthcare facilities (e.g., farms) seem to be low (*9*).

The nonspecificity of clinical definitions for MERS-CoV and the tendency of surveillance to focus on severe cases suggest that the prevalence of mild or asymptomatic infection cannot be estimated from case-based clinical surveillance alone (*10*). Mild or asymptomatic cases have been identified from contact tracing of laboratory-confirmed case-patients in several countries, including Saudi Arabia, the United Arab Emirates, Qatar, and South Korea (*11**–**16*).

In early October 2015, a cluster of MERS-CoV infections was identified among expatriate women working for a women-only university in Riyadh, Saudi Arabia. At the time the outbreak investigation was initiated, Kingdom of Saudi Arabia (KSA) Ministry of Health officials had identified 8 MERS case-patients by reverse transcription PCR (RT-PCR) (*17*); all patients were epidemiologically linked through their place of residence, a dormitory that housed expatriate women. Two additional laboratory-confirmed cases were identified among healthcare workers who had been exposed to the first case-patient, who had sought treatment at a medical clinic near the residence (*17*).

As part of this outbreak investigation, we conducted a molecular and seroepidemiologic study of the residents of an expatriate dormitory where the initial case-patients lived. Our goal was to describe and characterize the outbreak, determine potential source(s) of the outbreak, estimate the extent of MERS-CoV infection among residents, and evaluate risk factors for infection among residents.

## Methods

### Selection and Recruitment of Study Participants

We used the MERS-CoV standardized serologic investigation protocol developed by WHO and the Consortium for the Standardization of Influenza Seroepidemiology (*18*) and adapted it to the context of this outbreak. All 828 residents of the women-only expatriate dormitory in Riyadh were informed of the purpose of the outbreak investigation by KSA Ministry of Health official field teams and asked in person to participate. The KSA Ministry of Health, WHO, and Institut Pasteur field teams provided information sessions about the study and about MERS-CoV. The response team established a nursing station within the residential compound and assigned 2 nurses to reside within the compound to follow up with exposed persons and keep a log of any medical complaints from the residents throughout the outbreak period. Because this outbreak investigation was part of a public health response, it was not considered by the KSA Ministry of Health, Institut Pasteur, or The University of Hong Kong to be research that was subject to review by an institutional review board. As such, written informed consent was not required.

Included in the investigation were all residents of the dormitory who orally provided consent for completion of a questionnaire; collection of a nasopharyngeal or oropharyngeal swab sample, or both; and collection of a blood sample for serologic testing. Exclusion criteria included being <16 years of age at the time of recruitment, having any contraindication to venipuncture, or both.

The interviewers were trained to use the data collection forms developed for this investigation; because most residents were from the Philippines, the questionnaire was translated into Tagalog (Appendix). Each question was read aloud to women in groups of 15–25 in the dormitory while they filled in the questionnaire by hand. A subset of more sensitive questions was administered one-on-one by a member of the investigation team over the course of the 3-day field investigation. Before study implementation, frontline staff, including all outbreak investigation personnel, were trained with regard to infection control procedures, including proper hand hygiene and the correct use of respiratory face masks, to minimize their own risk for infection when in close contact with patients during home visits and elsewhere and to minimize the potential risk for MERS-CoV transmission between participants or between households.

### Specimen Collection and Testing for MERS-CoV

Any participant who reported respiratory symptoms during the initial investigation (October 19–28, 2015) or during a 14-day follow-up period (after last contact with a confirmed/suspected MERS-CoV patient) was immediately isolated, and nasopharyngeal/oropharyngeal swab samples were collected and tested for MERS-CoV by RT-PCR. RT-PCR testing of human biological specimens was conducted at the Riyadh Regional Laboratory by use of standardized RT-PCR methods for MERS-CoV testing (*19*). Any participants with a positive MERS-CoV result by RT-PCR according to WHO criteria (*10*) were reported to WHO under the requirements of the International Health Regulations (2005) (https://www.who.int/ihr/9789241596664/en).

On November 1–2, 2015, a total of 5 mL of blood was collected from consenting residents of the compound. The blood was collected in a serum collection tube according to standard procedures and labeled with a coded identification number linked to the data collection forms. Transport of specimens within national borders complied with the applicable national regulations of Saudi Arabia. International transport of MERS-CoV specimens followed applicable international regulations (*20*).

Serologic assays used to detect and confirm seropositivity in the serum samples were MERS-CoV S1 IgG ELISA (EUROIMMUN EI 2604–9601G kit, https://www.euroimmun.com), MERS-CoV spike pseudoparticle neutralization test (ppNT), and 90% plaque-reduction neutralization test (PRNT_90_). Serologic testing for MERS-CoV antibodies was conducted at the University of Hong Kong, as previously described (*21*). All serum samples were screened by MERS-CoV S1 ELISA, and positive or equivocal samples were further tested by ppNT and PRNT_90_. Serologic results were interpreted as positive if PRNT_90_ or ppNT titer for either the first or second serum specimen was >1:20.

### Statistical Analyses

We entered all data for analysis in the entry form in Epi Info 3.5.4 (https://www.cdc.gov/epiinfo) and exported it to statistical software Stata 14 (https://www.stata.com). We estimated risk factors for infection among case-patients and non–case-patients (risk ratios [RRs] and 95% CIs) and within a nested case–control study (odds ratios [ORs] and 95% CIs) by restricting analyses to residents living in villas in which laboratory-confirmed cases had been identified.

## Results

The first patient in this cluster who had laboratory-confirmed MERS was a 27-year-old woman who worked as a janitor in a women-only university in Riyadh. She reported experiencing dry cough and fatigue on October 1, 2015; she sought care at a private healthcare clinic on October 4 and was provided treatment and sent home the same day. On October 7, after signs and symptoms worsened to include fever, shortness of breath, productive cough, and signs of pneumonia, she again sought care in the same healthcare clinic, and a diagnosis of MERS was suspected. On October 8, a nasopharyngeal sample was collected and the patient was transferred to a public hospital in Riyadh, designated for isolation and treatment of MERS patients. MERS-CoV infection was confirmed on October 9. A second case in this cluster has recently been described (*22*).

**Figure 1:**
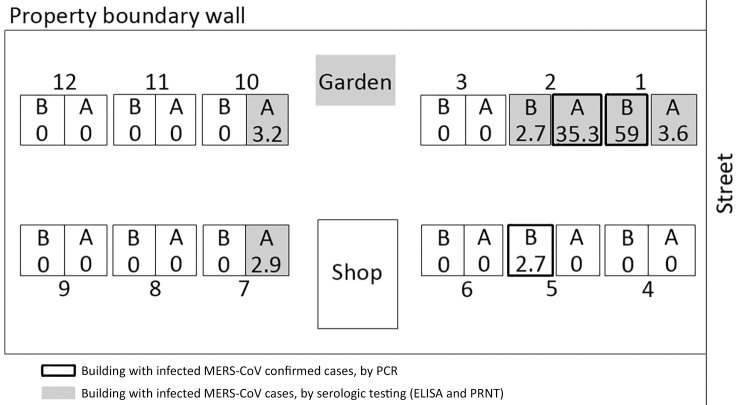
Schematic of expatriate dormitory (the residence, buildings 1–12) and MERS-CoV infection attack rates (IARs), Riyadh, Saudi Arabia, 2015. Each building contained 2 villas on 3 floors. The distance between buildings is ≈5 m. During the initial investigation (October 2015), 8 residents were positive for MERS-CoV by PCR (indicated by black boxes); they lived in buildings 1B, 2A, and 5B. A vegetable garden separated buildings 3 and 10, and a convenience store (shop) separated buildings 6 and 7. IARs are shown as percentages inside each villa. MERS-CoV, Middle East respiratory syndrome coronavirus; PRNT, plaque-reduction neutralization test.

The first patient resided in an enclosed, women-only, expatriate dormitory composed of 24 villas (Figure 1). Each villa is a 3-story building with 7 bedrooms (2 on the ground floor, 3 on the first floor, and 2 on the second floor) and is inhabited by 24–50 women. On inspection of the living quarters, the field team found that most of the windows in the bedrooms were closed and sealed and that ventilation within the bedrooms was poor. Initial open-ended interviews with some residents informed the study team that residents shared the same kitchen and dining room within the villa but did not typically eat together or share food at mealtimes. There were no designated social spaces; however, residents reported gathering around laptops to watch movies together.

A total of 828 women who lived in the residence complex were included in the seroepidemiologic study; none of the eligible women refused to participate. All participants were female, and median age was 35.1 (26.6–41.3) years. None were Saudi Arabia nationals; they were from the Philippines (84.6%), Sri Lanka (6.4%), Indonesia (2.9%), Nepal (1.6%), and India (1.1%) (Table 1). A total of 49 participants (1 case-patient and 48 non–case-patients) reported having >1 chronic condition (e.g., asthma, diabetes, heart disease, hypertension, breast cancer) (Table 1). The MERS case-patient reported having asthma; among non–case-patients, the most common chronic conditions reported were asthma (31%), diabetes (25%), and hypertension (18%).

**Table 1:** Demographic characteristics of patients with and without MERS-CoV infection, Riyadh, Saudi Arabia, 2015*

Characteristics	All participants, no. (%), n = 828	Case-patients, no. (%), n = 19†	Non–case-patients, no. (%), n = 809
Sex			
F	814/814 (100)‡	19/19 (100)‡	795/795 (100)‡
M	0	0	0

Nationality	779	19	760
Filipino	659 (84.6)	19 (100)	640 (84.2)
Sri Lankan	50 (6.4)	0	50 (6.6)
Nepali	12 (1.5)	0	12 (1.6)
Bangladeshi	28 (3.6)	0	28 (3.7)
Indonesian	22 (2.8)	0	22 (2.9)
Indian	8 (1.0)	0	8 (1.0)

Highest level of education reached	779	19	761
Primary school	80 (10.3)	1 (5.3)	79 (10.4)
High school	377 (48.4)	10 (52.6)	368 (48.4)
University/diploma	234 (30.0)	4 (21.1)	230 (30.3)
Postgraduate degree	77 (9.9)	4 (21.1)	73 (9.6)
No education	11 (1.4)	0	11 (1.4)

Primary occupation	770	19	751
Women-only university	378(49.1)	17 (89.5)	361 (48.1)
Public university	12 (1.6)	0	12(1.6)
Hospital A	32 (4.2)	0	32 (4.3)
Hospital B	238 (30.9)	2 (10.5)	236 (31.4)
Hospital C	54 (7.0)	0	54 (7.2)
Hospital D	56 (7.3)	0	56 (7.5)

Secondary occupation	83/805 (10.3)	3 (15.8)	80 (10.7)
Hospital A	NA	2 (10.5)	17 (2.3)
Hospital D	NA	1 (5.3)	10 (1.3)
Other (health club)	NA	0	53 (7.0)

Any underlying medical conditions	49/780 (6.3)	1 (5.0)	48/761 (6.3)
Regularly smoke (% daily)	10/773 (1.3)	1/19 (5.6)	9/755 (1.2)
Current chronic conditions§	49/780 (6.3)	1/19 (5.3)	48/761 (6.3)

*Median age (interquartile range): for all, 35.1 (26.6–41.3) years; for case-patients, 29.8 (28–37.2) years; for non–case-patients, 35.2 (29.6–41.4) years. CoV, coronavirus; MERS, Middle East respiratory syndrome; NA, not applicable. †Molecular or serologic evidence of MERS-CoV infection.  ‡Denominator indicates the number of women who answered the question. §Included asthma, diabetes, heart disease, hypertension, and breast cancer.

In terms of occupation, almost half (49.1%) of participants reported working at the women-only university in Riyadh, including 17 (89.5%) of the MERS case-patients (Table 1). Participants reported working in 1 of 4 hospitals as either their primary or secondary occupation (Table 1).

**Figure 2:**
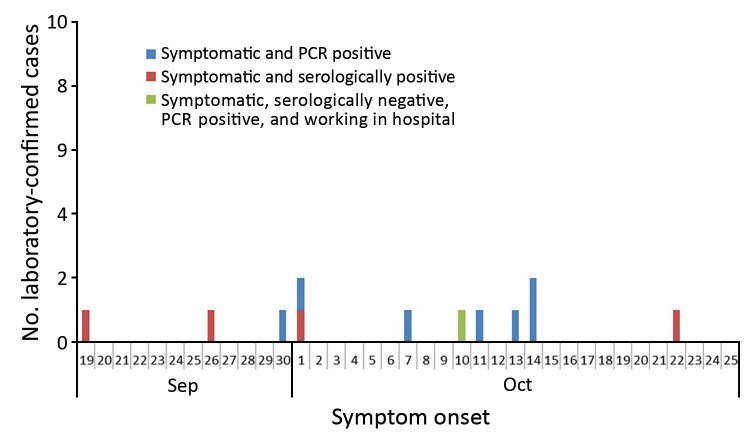
Epidemiologic curve for symptomatic laboratory-confirmed case-patients with Middle East respiratory syndrome coronavirus infection, Riyadh, Saudi Arabia, 2015. The curve includes only the 12 case-patients for whom symptom onset was reported, not the 7 case-patients for whom infection was serologically confirmed but no symptoms were reported in the preceding 4 weeks.

Contact tracing of the initial patient and molecular and serologic laboratory test results identified an additional 18 MERS-CoV infections (Figure 2; Table 2). Of the 19 total case-patients, 12 (63.2%) were from villa 2A; 2 (10.5%) were from a facing villa (1B); and 1 case (5.3%) was reported from each of 5 villas either close to the mostly affected villa (2A) or 2 other villas (10A and 7A) populated with residents from the Philippines (Figure 1).

**Table 2:** Characteristics of the MERS-CoV–positive participants identified from molecular and serologic assay results, Riyadh, Saudi Arabia, 2015*

Age, y	Bldg no.	Signs/ symptoms†	Symptom onset date	RT-PCR‡	Serologic test								
					SI ELISA			ppNT			PRNT_90_		Serologic test result§
					First sample	Second sample		First sample	Second sample		First sample	Second sample	
23	1B	Yes	Oct 11	+	1.586	0.523		80	20		20	10	+
28	5B	Yes	Oct 14	+	2.225	NA		80	NA		40	NA	+
29	2A	Yes	Oct 13	+	1.181	NA		20	NA		10	NA	+
29	2A	Yes	Oct 14	+	4.57	NA		160	NA		80	NA	+
28	2A	Yes	Oct 1	+	3.154	2.741		160	160		40	40	+
26	2A	Yes	Oct 7	+	3.154	NA		160	NA		40	NA	+
39	2A	Yes	Sep 30	+	1.553	NA		40	NA		20	NA	+
53	2A	No	NS	+	4.242	NA		160	NA		80	NA	+
41	1B	No	NS	NA	1.311	0.33		20	10		10	<10	+
37	2A	Yes	Oct 10	–	1.214	0.569		40	20		10	<10	+
30	2A	Yes	Oct 22	–	0.759	0.605		20	20		0	<10	+
24	2A	Yes	Oct 1	–	1.422	NA		80	NA		20	NA	+
32	2A	Yes	Sep 26	–	3.381	1.012		80	20		20	10	+
28	2A	Yes	Sep 19	–	1.999	1.654		40	40		10	20	+
30	1A	No	NS	NA	3.295	1.496		40	20		10	<10	+
36	2B	No	NS	–	1.419	NA		20	NA		20	NA	+
42	7A	No	NS	NA	0.576	NA		01:10	NA		20	NA	+
37	10A	No	NS	NA	1.115	NA		0.097222	NA		80	NA	+
45	2A	No	NS	–	1.111	0.563		20	20		<10	<10	+

*First samples collected November 13, 2015; second samples collected March 22, 2015. Bldg, building; CoV, coronavirus; MERS, Middle East respiratory syndrome; NA, not available/not collected; NS, no signs/symptoms reported; ppNT, pseudoparticle neutralization test; PRNT_90_, 90% plaque-reduction neutralization test; RT-PCR, reverse transcription PCR; +, positive; –, negative.  †Self-reported or observed signs/symptoms in the 14 d before epidemiologic interview. ‡According to World Health Organization criteria (http://www.who.int/csr/disease/coronavirus_infections/mers-laboratory-testing). §Serologic test result was defined as positive if either PRNT_90_ or ppNT titers were >20. S1 ELISA results are shown for information only; they were not used in designating infection status.

Among the 8 MERS-CoV cases positive by PCR, 8 were also serologically positive for MERS-CoV (Table 2). According to PRNT_90_ or ppNT serology results for either the first or second serum sample, an additional 11 persons were serologically positive for MERS-CoV infections. Therefore, a total of 19 of the 828 dormitory residents had evidence of MERS-CoV infection by molecular or serologic testing or both; the infection attack rate [IAR] for the cohort was 2.3%.

Of the 9 patients from whom a second sample was collected in March 2016, a total of 8 had ppNT titers of >1:20, and only 2 of these had PRNT_90_ titers of >1:20. For 2 of these 8 patients, ppNT indicated a >4-fold fall in antibody titer; for the others, ppNT antibody levels remained within 2-fold that of the initial serum sample.

Bivariate analyses indicated significant assocations between MERS and the following risk factors: having direct contact with a known MERS patient (RR 10.9, 95% CI 6.7–17.6); sharing a bedroom (RR 25.5, 95% CI 10.3–63.1), kitchen (RR 15.5, 95% CI 5.4–44.2), bathroom (RR 25.5, 95% CI 10.3–63.1), meal (RR 19.4, 95% CI 7.5–50.3), or transportation vehicle (RR 11.8, 95% CI 4.9–28.5); and having indirect contact with a known patient (RR 15.5, 95% CI 5.4–44.2) (Table 3). The presence of a chronic condition did not vary by MERS infection status. According to multivariate analyses, direct contact with a known MERS patient (OR 27.6, 95% CI 8.4–91.0) and sharing a bedroom with a MERS patient (OR 5.7, 95% CI 1.5–22.5) remained statistically significant. Having a functioning air conditioner in the bedroom was protective (OR 0.15, 95% CI 0.03–0.82). None of the women reported traveling outside of Saudi Arabia in the 14 days before symptom onset (data not shown).

**Table 3:** Bivariate analyses of reported exposures to known MERS patient, including overall cohort, Riyadh, Saudi Arabia, 2015*

Reported exposure	Case-patients, no. (%), n = 19	Non–case-patients, no. (%), n = 809	p value†	RR (95% CI)
Direct contact with known (symptomatic) MERS-CoV case-patient	11 (57.9)	43 (5.3)	<0.001	10.9 (6.7–17.6)
Shared bedroom with known case-patient	6 (31.6)	10 (1.2)	<0.001	25.5 (10.3–63.1)
Shared kitchen with known case-patient	4 (21.1)	11 (1.4)	<0.001	15.5 (5.4–44.2)
Shared bathroom with known case-patient	6 (31.6)	10 (1.2)	<0.001	25.5(10.3–63.1)
Shared meal with known case-patient	5 (26.3)	11 (1.4)	<0.001	19.4 (7.5–50.3)
Shared transportation to/from place of employment with known case-patient	5 (26.3)	18 (2.2)	<0.001	11.8 (4.9–28.5)
Reported nondirect contact with case-patient‡	4 (21.1)	11 (1.4)	<0.001	15.5 (5.4–44.2)

*CoV, coronavirus; MERS, Middle East respiratory syndrome; RR, risk ratio. †By χ^2^ test. ‡No physical contact, nonphysical contact (including talk to the known case-patient).

## Discussion

This study details the comprehensive investigation of a cluster of MERS cases reported outside a healthcare-associated or camel industry–associated occupational setting. In this women-only, expatriate worker dormitory in Riyadh, Saudi Arabia, the overall IAR of 2.3% is similar to that found in a household contact study conducted in 2014 (IAR of 4.3%) (*9*). However, in this outbreak, the residential setting was more crowded than typical single-family households. Although we found the IAR in some villas to be low, we identified IARs as high as 35.3% (12/34) in 1 villa (2A), probably because of the exceptionally crowded living and sleeping conditions. Within this villa, 12 women were infected with MERS-CoV but only 10 reported any symptoms. Rates of IAR were not affected by the presence or absence of underlying conditions or the median age of residents by villa.

This study identified the independent risk factors for infection to be direct contact and sharing a bedroom with a MERS patient. Findings from other serologic studies have been similar (*23*). We hypothesize that the increased human-to-human transmission within villas resulted from the clustering of the women’s activities. For example, the same women who lived together typically ate and socialized together, worked together, and traveled to and from work together. These activities added to the likelihood of intense direct physical contact among the women and probably led to limited but effective human-to-human transmission within their residence.

Globally, the extent of human-to-human transmission outside of healthcare facilities is uncertain, and whether MERS-CoV has the potential for sustained community transmission is unclear. Transmission among family members seems to be limited but can be amplified in healthcare settings (*24*,*25*) among persons with underlying medical conditions and to healthcare workers. Contributors to propagation of MERS-CoV infection in healthcare facilities include aerosol-generating procedures such as intubation, suction, and collection of nasopharyngeal swabs (*26*). Compared with the total number of MERS-CoV infections reported to WHO to date, patients in our study cohort were significantly younger (median age 32 vs. 52 years, respectively), healthier (6.3% vs. 41.0% reporting >1 chronic condition), and more likely to be female (0 vs. 68.1% male) (*27*).

Healthcare staff can prevent human-to-human transmission of MERS-CoV through stringent adherence and implementation of detailed and clear protocols for standard, droplet, and aerosol infection prevention and control (IPC) measures among the various persons within a healthcare setting (i.e., healthcare workers, patients, and visitors) (*28*). Such IPC measures were not followed by the inhabitants of the dormitory in this study.

Although we were able to rule out a connection to dromedary camels, we were not able to specifically determine the source of this outbreak. Of the 19 laboratory-confirmed case-patients, 17 reported working at the same women-only university in Riyadh and the other 2 worked primarily as cleaners at the same healthcare facility in Riyadh (hospital B). Of these 19 case-patients, 3 also reported having a secondary place of employment, including working as cleaners at 2 other hospitals in Riyadh (hospitals A and D). We hypothesize that 1 of the 19 infected women identified in this investigation may have been exposed to and infected with MERS-CoV while working as a cleaner in a healthcare facility where persons with undiagnosed MERS had been cared for. In August 2015, hospital B, reportedly the primary occupation location for 2 women who were MERS-CoV positive according to PCR, was the location of a small cluster of laboratory-confirmed MERS cases (n = 5). Unfortunately, viral genetic sequencing was conducted on only 1 of those patients (*22*); without further epidemiologic and sequencing data from other patients in this cluster, or from the laboratory-confirmed patients in the small cluster in hospital B in August 2015, we cannot surmise further.

The time lag between identification of MERS patients in hospital B in August 2015 and the timing of this outbreak in October 2015 suggests that persons with subclinical cases may have been in or working in this hospital during August–October 2015; however, because testing for MERS-CoV in Saudi Arabia was substantial (*29*), missing symptomatic cases was unlikely. A subject of some debate and recent focus has been the potential role of mildly symptomatic or asymptomatic infections and possible environmental contamination in the spread of MERS-CoV in healthcare facilities (*22*,*30**–**33*). The rapid initiation of this investigation and use of an existing protocol (*34*) (developed for such use after the rapid isolation of close contacts regardless of the development of symptoms and the implementation of a no-fly policy among residents of the compound until the full 14-day follow-up was completed) probably limited further human-to-human transmission inside and potentially outside of Saudi Arabia.

Our study highlights the potential role of healthcare workers not responsible for direct patient care (e.g., hospital cleaners) in the spread of MERS-CoV. Often, hospital cleaning staff may be from other countries, may speak several languages, and may be missed by efforts to increase IPC specific to MERS-CoV. Specific MERS-CoV IPC training should be directed to cleaning staff in healthcare facilities, in addition to healthcare providers, in appropriate languages, particularly to protect them from infection and from facilitating virus spread within the healthcare facility.

For the 8 women with RT-PCR–confirmed infection, antibody titers ranged from 1:10 to 1:80 by PRNT and from 1:20 to 1:160 by ppNT. For 9 of the 19 women with confirmed evidence of infection by RT-PCR, serologic testing, or both, for whom follow-up serum samples were available 3 months after the putative exposure, 7 women had PRNT titers of <1:20 and 1 woman had ppNT titers of <1:20. Thus, the ppNT antibody test was somewhat more sensitive for detecting evidence of past infection. A ppNT titer of 1:20 is therefore an optimal indicator of past infection in seroepidemiologic assays. The ppNT, although more sensitive, correlated well with PRNT among persons with RT-PCR–confirmed MERS-CoV infection (*35*) and was uniformly negative in serum from persons in areas where MERS-CoV is not endemic (e.g., Hong Kong [*36*]). For this study, we categorized those without RT-PCR evidence of MERS-CoV infection but PRNT or ppNT antibody titers >1:20 as being MERS-CoV infected.

Of the 8 women who had RT-PCR–confirmed infection, 2 were asymptomatic, as were 6 of the 11 women whose diagnosis was made solely by serologic testing. Serologic studies of cohorts of patients positive for MERS-CoV by RT-PCR have shown that milder disease and asymptomatic infections may not be associated with detectable serologic responses (*37*). Thus, our serologic testing probably underestimates the true number of MERS-CoV infections that may have occurred. However, our data provide evidence that even asymptomatic infections can sometimes lead to detectable serologic responses and that such investigations are useful. Furthermore, the serologic results at 5 months after putative exposure show evidence of antibody titers waning to below diagnostic limits in some patients but also show that antibodies may remain detectable in others. This information is useful when interpreting seroepidemiologic studies in high-risk populations.

Our study had several limitations. Because of multicollinearity of the exposure variables (*38*), the accuracy of individual predictors may be compromised. The lack of collection of acute blood samples during the outbreak limited our ability to detect seroconversion. In addition, we were not able to conduct sequencing for patients of this outbreak and therefore were not able to use this information to potentially confirm that all 19 infected women acquired their infection from a common source or to identify the source of the outbreak.

The rapid initiation of contact tracing, isolation, and subsequent investigation probably contributed to the quick halt of human-to-human transmission in this outbreak. On the basis of the possible source of infection, to reduce secondary human-to-human transmission outside the occupational setting, our study indicates that IPC measures introduced in healthcare facilities should focus on not only healthcare personnel but also those working within the wider facility, including cleaners.

Appendix Questionnaire used in study of transmissibility of MERS-CoV infection in closed setting, Riyadh, Saudi Arabia, 2015.

## References

[1] Who Mers-Cov Research Group. State of knowledge and data gaps of Middle East respiratory syndrome coronavirus (MERS-CoV) in humans. PLoS Curr. 2013;5:ecurrents.outbreaks.0bf719e352e7478f8ad85fa30127ddb8. 10.3201/eid1911.131172 24270606 PMC3828229

[2] World Health Organization. Middle East respiratory syndrome–coronavirus– update: 29 May 2013 [cited 2013 May 30]. http://www.who.int/csr/don/2013_05_29_ncov

[3] Zaki AM, van Boheemen S, Bestebroer TM, Osterhaus AD, Fouchier RA. Isolation of a novel coronavirus from a man with pneumonia in Saudi Arabia. N Engl J Med. 2012;367:1814–20. 10.1056/NEJMoa1211721 23075143

[4] Hijawi B, Abdallat M, Sayaydeh A, Alqasrawi S, Haddadin A, Jaarour N, et al. Novel coronavirus infections in Jordan, April 2012: epidemiological findings from a retrospective investigation. East Mediterr Health J. 2013;19(Suppl 1):S12–8. 10.26719/2013.19.supp1.S12 23888790

[5] Aguanno R, ElIdrissi A, Elkholy AA, Ben Embarek P, Gardner E, Grant R, et al.; FAO-OIE-WHO MERS Technical Working Group. MERS: Progress on the global response, remaining challenges and the way forward. Antiviral Res. 2018;159:35–44. 10.1016/j.antiviral.2018.09.002 30236531 PMC7113883

[6] Ben Embarek PK, Van Kerkhove MD. Middle East respiratory syndrome coronavirus (MERS-CoV): current situation 3 years after the virus was first identified. Wkly Epidemiol Rec. 2015;90:245–50. 25980038

[7] Drosten C, Muth D, Corman VM, Hussain R, Al Masri M, HajOmar W, et al. An observational, laboratory-based study of outbreaks of middle East respiratory syndrome coronavirus in Jeddah and Riyadh, kingdom of Saudi Arabia, 2014. Clin Infect Dis. 2015;60:369–77. 10.1093/cid/ciu812 25323704 PMC4303774

[8] Ki M. 2015 MERS outbreak in Korea: hospital-to-hospital transmission. Epidemiol Health. 2015;37:e2015033. 10.4178/epih/e2015033 26212508 PMC4533026

[9] Drosten C, Meyer B, Müller MA, Corman VM, Al-Masri M, Hossain R, et al. Transmission of MERS-coronavirus in household contacts. N Engl J Med. 2014;371:828–35. 10.1056/NEJMoa1405858 25162889

[10] World Health Organization. Middle East respiratory syndrome coronavirus (MERS-CoV) [cited 2019 Jul 30]http://www.who.int/emergencies/mers-cov

[11] Al Hosani FI, Pringle K, Al Mulla M, Kim L, Pham H, Alami NN, et al. Response to emergence of Middle East respiratory syndrome coronavirus, Abu Dhabi, United Arab Emirates, 2013–2014. Emerg Infect Dis. 2016;22:1162–8. 10.3201/eid2207.160040 27314227 PMC4918155

[12] Alraddadi B, Bawareth N, Omar H, Alsalmi H, Alshukairi A, Qushmaq I, et al. Patient characteristics infected with Middle East respiratory syndrome coronavirus infection in a tertiary hospital. Ann Thorac Med. 2016;11:128–31. 10.4103/1817-1737.180027 27168861 PMC4854059

[13] World Health Organization. Disease outbreak news [cited 2019 Jul 30]. http://www.who.int/csr/don/22-june-2016-mers-saudi-arabia

[14] Memish ZA, Zumla AI, Assiri A. Middle East respiratory syndrome coronavirus infections in health care workers. N Engl J Med. 2013;369:884–6. 10.1056/NEJMc1308698 23923992

[15] Oboho IK, Tomczyk SM, Al-Asmari AM, Banjar AA, Al-Mugti H, Aloraini MS, et al. 2014 MERS-CoV outbreak in Jeddah—a link to health care facilities. N Engl J Med. 2015;372:846–54. 10.1056/NEJMoa1408636 25714162 PMC5710730

[16] Moon SY, Son JS. Infectivity of an asymptomatic patient with Middle East respiratory syndrome coronavirus infection. Clin Infect Dis. 2017;64:1457–8. 10.1093/cid/cix170 28444154 PMC7108064

[17] Kingdom of Saudi Arabia Ministry of Health. Weekly monitor MERS-CoV. 3 November 2015 [cited 2019 Jul 24]. https://www.moh.gov.sa/en/CCC/Documents/Volume-2-Issue-11-Tuesday-March-15-2016.pdf

[18] World Health Organization. Seroepidemiological investigation of contacts of Middle East respiratory syndrome coronavirus (MERS-CoV) patients [cited 2019 Aug 1]. https://www.who.int/csr/disease/coronavirus_infections/who-close-non-hcw-contact-protocol-mers-cov.docx?ua=1

[19] Command and Control Center SAB. Kingdom of Saudi Arabia Ministry of Health. Middle East respiratory syndrome coronavirus guidelines for healthcare professionals [cited 2019 Aug 1]. https://www.moh.gov.sa/CCC/healthp/regulations/Documents/MERS-CoV%20Guidelines%20for%20Healthcare%20Professionals%20-%20May%202018%20-%20v5.1%20%281%29.pdf

[20] World Health Organization. Guidance on regulations for the transport of infectious substances 2015–2016 [cited 2019 Jun 5]. http://www.who.int/ihr/publications/who_hse_ihr_2015.2

[21] Choe PG, Perera RAPM, Park WB, Song K-H, Bang JH, Kim ES, et al. MERS-CoV antibody responses 1 year after symptom onset, South Korea, 2015. Emerg Infect Dis. 2017;23:1079–84. 10.3201/eid2307.170310 28585916 PMC5512479

[22] Al-Abdely HM, Midgley CM, Alkhamis AM, Abedi GR, Tamin A, Binder AM, et al. Infectious MERS-CoV isolated from a mildly ill patient, Saudi Arabia. Open Forum Infect Dis. 2018;5:ofy111. 10.1093/ofid/ofy111 30294617 PMC6016420

[23] Arwady MA, Alraddadi B, Basler C, Azhar EI, Abuelzein E, Sindy AI, et al. Middle East respiratory syndrome coronavirus transmission in extended family, Saudi Arabia, 2014. Emerg Infect Dis. 2016;22:1395–402. 10.3201/eid2208.152015 27191038 PMC4982159

[24] Breban R, Riou J, Fontanet A. Interhuman transmissibility of Middle East respiratory syndrome coronavirus: estimation of pandemic risk. Lancet. 2013;382:694–9. 10.1016/S0140-6736(13)61492-0 23831141 PMC7159280

[25] Cauchemez S, Fraser C, Van Kerkhove MD, Donnelly CA, Riley S, Rambaut A, et al. Middle East respiratory syndrome coronavirus: quantification of the extent of the epidemic, surveillance biases, and transmissibility. Lancet Infect Dis. 2014;14:50–6. 10.1016/S1473-3099(13)70304-9 24239323 PMC3895322

[26] Hui DS, Azhar EI, Kim Y-J, Memish ZA, Oh MD, Zumla A. Middle East respiratory syndrome coronavirus: risk factors and determinants of primary, household, and nosocomial transmission. Lancet Infect Dis. 2018;18:e217–27. 10.1016/S1473-3099(18)30127-0 29680581 PMC7164784

[27] World Health Organization. WHO MERS global summary and assessment of risk: August 2018 [cited 2019 Jun 5]. https://www.who.int/csr/disease/coronavirus_infections/risk-assessment-august-2018.pdf

[28] World Health Organization. Infection prevention and control during health care for probable or confirmed cases of Middle East respiratory syndrome coronavirus (MERS-CoV) infection [cited 2019 Jun 5]. https://www.who.int/csr/disease/coronavirus_infections/ipc-mers-cov

[29] Saeed AA, Abedi GR, Alzahrani AG, Salameh I, Abdirizak F, Alhakeem R, et al. Surveillance and testing for Middle East respiratory syndrome coronavirus, Saudi Arabia, April 2015–February 2016. Emerg Infect Dis. 2017;23:682–5. 10.3201/eid2304.161793 28322710 PMC5367404

[30] Van Kerkhove MD, Peiris MJS, Malik MR, Ben Embarek P. Interpreting results from environmental contamination studies of Middle East respiratory syndrome coronavirus. Clin Infect Dis. 2016;63:1142. 10.1093/cid/ciw478 27432840 PMC7108072

[31] Kim S-H, Chang SY, Sung M, Park JH, Bin Kim H, Lee H, et al. Extensive viable Middle East respiratory syndrome (MERS) coronavirus contamination in air and surrounding environment in MERS outbreak units. Clin Infect Dis. 2016;63:363–9. 10.1093/cid/ciw239 27090992 PMC7108054

[32] Bin SY, Heo JY, Song M-S, Lee J, Kim E-H, Park S-J, et al. Environmental contamination and viral shedding in MERS patients during MERS-CoV outbreak in South Korea. Clin Infect Dis. 2016;62:755–60. 10.1093/cid/civ1020 26679623 PMC7108026

[33] van Doremalen N, Bushmaker T, Munster VJ. Stability of Middle East respiratory syndrome coronavirus (MERS-CoV) under different environmental conditions. Euro Surveill. 2013;18:20590. 10.2807/1560-7917.ES2013.18.38.20590 24084338

[34] World Health Organization. Assessment of potential risk factors of Middle East respiratory syndrome coronavirus (MERS-CoV) infection among health care personnel in a health care setting [cited 2019 Jan 1]. https://www.who.int/csr/disease/coronavirus_infections/who-generic_healthcare-mers-seroepi-investigation.docx

[35] Park SW, Perera RA, Choe PG, Lau EH, Choi SJ, Chun JY, et al. Comparison of serological assays in human Middle East respiratory syndrome (MERS)-coronavirus infection. Euro Surveill. 2015;20:30042. 10.2807/1560-7917.ES.2015.20.41.30042 26538277

[36] Perera RA, Wang P, Gomaa MR, El-Shesheny R, Kandeil A, Bagato O, et al. Seroepidemiology for MERS coronavirus using microneutralisation and pseudoparticle virus neutralisation assays reveal a high prevalence of antibody in dromedary camels in Egypt, June 2013. Euro Surveill. 2013;18:20574. 10.2807/1560-7917.ES2013.18.36.20574 24079378

[37] Ko JH, Müller MA, Seok H, Park GE, Lee JY, Cho SY, et al. Serologic responses of 42 MERS-coronavirus-infected patients according to the disease severity. Diagn Microbiol Infect Dis. 2017;89:106–11. 10.1016/j.diagmicrobio.2017.07.006 28821364 PMC7127792

[38] Vatcheva KP, Lee M, McCormick JB, Rahbar MH. Multicollinearity in regression analyses conducted in epidemiologic studies. Epidemiology (Sunnyvale). 2016;6:227. 10.4172/2161-1165.1000227 27274911 PMC4888898

