# Supplementary material for: Transmissibility of MERS-CoV Infection in Closed Setting, Riyadh, Saudi Arabia, 2015
Source: Emerg Infect Dis. 2019 Oct;25(10):1802–9. doi: 10.3201/eid2510.190130 (PMC6759265; doi:10.3201/eid2510.190130)
Supplement: Appendix — Questionnaire used in study of transmissibility of MERS-CoV infection in closed setting, Riyadh, Saudi Arabia, 2015. [file 19-0130-Techapp-s1.pdf]

Article DOI: https://doi.org/10.3201/eid2510.190130

Transmissibility of MERS-CoV Infection in a Closed Setting, Riyadh, Saudi Arabia, 2015

Appendix

Outbreak Investigation Questionnaire

The following questionnaire was administered in groups of 15 participants by a trained interviewer from the Ministry of Health or Institut Pasteur. The interviewer read each question aloud while the participants wrote their answers directly into the questionnaire.

MERS-CoV Outbreak Investigation Questionnaire

GENERAL INFORMATION

PANGKALAHATANG IMPORMASYON

1. Subject ID: ___ ___ ___ ___ ___ ___ ___ ___

Numero ng ID: ___ ___ ___ ___ ___ ___ ___ ___

2. Subject Name: First name ________________ Surname________________

Panglan: ___________________________ Apelyido: _________________

3. **Date of interview (*dd/mm/yyyy*): ____/____/____**

Petsa ng pakikipanayam o interbyu (araw/buwan/taon) _____/_____/_____

4. **Location of Interview** (*Region*, *City*, *Province*):

____

Lugar ng pakikipanayam (Rehiyon/Syudad/Probinsya):

____

5. **Language used for interview:** ☐ English ☐ Filipino ☐ Other, please specify **______**

Gamit na salita sa pakikipanayam: ☐ English ☐ Tagalog/Filipino ☐ At iba pa, (tukuyin)**__________**

6. **Gender (tick one):** ☐ Female ☐ Male

Kasarian (lagyan ng tsek ang isa): ☐Babae ☐ Lalake

7. **Place of primary residence of subject (address):**

____

Lugar ng pangunahing paninirahan ng paksa/pasyente (address):

____

8. How long have you lived in this complex? _____ months _____ year

Gaano katagal ka ng nanirahan sa complex na ito: ________buwan _________ taon

9. **Date of birth:** ____/____/_____ (mm/dd/yyyy)

Petsa ng kapanganakan: _____/_____/_____ (buwan/araw/taon)

10. **What is your current marital status?** ☐ Single ☐ Married ☐ Divorce ☐ Widowed

Ano ang iyong kasalukuyang katayuan civil? ☐ Single ☐ Kasal ☐ Diborsyado/Dibosyada ☐ Byudo/byuda

a. If you are married, does your husband live in KSA? ☐ Yes ☐ No

Kung ikaw ay may-asawa, ang iyong asawa ay nakatira sa KSA? ☐ Oo ☐ Hindi

b. If yes, where does he live? (location, city province) ______________________

Kung Oo, saan siya nakatira? (lokasyon, syudad/probinsya) ______________

c. If yes, how often do you visit your husband? ☐ More than once a week ☐ once a week ☐ once a month

Kung oo, gaano kadalas mo bisitahin ang iyong asawa? ☐ Higit sa isang beses sa isang lingo

☐minsan sa isang lingo

☐minsan sa isang buwan

11. What is your nationality? ☐ Filipino ☐ Indian ☐ Indonesian ☐ Sri Lankan ☐ Other _________

Ano ang iyong nasyonalidad? ☐ Filipino ☐ Indian ☐ Indonesian ☐ Sri Lankan ☐ At iba pa: _______

12. How long have you been living in Saudi Arabia? _________

Gaano katagal ka na naninirahan sa Saudi Arabia? _________

HOUSING EXPOSURES

13. What building do you live in ? _____________
Anong building ang iyong tinitirhan? ____________
14. What floor do you live on? ☐ Ground floor ☐ First floor ☐ Second floor
Sa anong floor ka nakatira? ☐ Palapag ☐Unang palapag ☐Pangalawang palapag

15. How many women live in your villa with you in total? ________ total

Gaano karaming mga kababaihan ang nakatira sa inyong villa? __________(lahat)

i. **Ground floor: _____** Palapag: _________

ii. **First floor: ________** Unang palapag:_______

iii. **Second floor: ______** Pangalawang palapag: _____

16. What is your bedroom number? ☐ One ☐ Two ☐ Three
Ano ang iyong bedroom number? ☐ Una/Isa ☐Panglawa/Dalawa ☐ Pangatlo/Tatlo
17. How many women share the bedroom with you? _______________ women
Gaano karaming mga kababaihan kasama/kabahagi mo sa kwarto? ___________babae
18. Do you have a bottom or top bunk? ☐ Top ☐ Bottom
Mayroon ba kayong isang ibaba o itaas bunk? ☐ Itaas ☐ Ibaba
18.1 Do you have a personal electronic fan in your bed space? ☐ Yes ☐ No
Mayroon ba kayong isang personal na elektronikong fan sa iyong kwarto? ☐ Meron ☐ Wala
18.2 Do you use a curtain on your bunk bed? ☐ Yes ☐ No
Gumagamit ka ba ng isang kurtina sa iyong bunk bed? ☐ Oo ☐ Hindi
19. Is there a window in your bedroom? ☐ Yes ☐ No
Mayroon bang bintana sa iyong kwarto? ☐ Meron ☐ Wala
19.1 f yes, is this window covered? ☐ Yes ☐ No
Kung Oo, may takip ba ang bintana na ito? ☐ Meron ☐ Wala
20. Is there a working air conditioner in your bedroom? ☐ Yes ☐ No
Mayroon bang gumaganang air conditioner sa iyong kwarto? ☐ Meron ☐ Wala

21. How many bathrooms are on your floor? ☐ 1 ☐ 2 ☐ 3

Ilan ang mga banyo sa iyong palapag(floor)? ☐ 1 ☐ 2 ☐ 3

21.1 How many women share your bathroom? ______ women ☐ Unknown

Gaano karaming mga babae ang gumagamit ng iyong banyo? _______babae

22. Is there a kitchen on your floor? ☐ Yes ☐ No

Mayroon bang isang kusina sa iyong palapag? ☐ Meron ☐ Wala

22.1 If not, which kitchen(s) do you use (check all that apply)?

☐ Ground floor ☐ First floor ☐ Second Floor

Kung wala, alin or saan (mga) kitchen ang iyong ginagamit (i-check ang lahat ng naaangkop)?

☐ Palapag ☐ Unang palapag ☐ Pangalawang palapag

22.2 How many women share the kitchen you use? ______ women ☐ Unknown

Gaano karaming mga babae ang gumagamit ng iyong kusina? __________

22.3 Where is your refrigerator? ☐Bedroom ☐Kitchen ☐both Bedroom and Kitchen

Saan ang iyong refrigerator? ☐Silid Tulugan ☐Kusina ☐Pareho Silid tulugan at Kusina

23. Is there a washing machine on your floor? ☐ Yes ☐ No

Mayroon bang isang washing machine sa iyong palapag? ☐ Meron ☐ Wala

23.1 Do you use the washing machine to clean your clothes? ☐ Yes ☐ No

Ginagamit mo ba ang washing machine upang linisin ang iyong mga damit?☐ Oo ☐ Hindi

23.2 If not, which washing machine do you use? ☐ Ground floor☐ First floor ☐ Second floor ☐ Don't use washing machine

Kung Hindi, ano o saan ang washing machine ang ginagamit mo? ☐ Palapag ☐ Unang Palapag ☐ Pangalawang palapag ☐ Hindi gumagamit ng washing machine

23.3 How many women share the washing machine you use? ________ women

Gaano karaming mga babae ang kashare mo sa ginagamit mong washing machine? _______babae

24. Have you seen other animals or pests at your home? ☐ YES ☐ NO

Mayroon bang mga hayop o mga peste sa iyong bahay? ☐ Meron ☐ Wala

24.1If yes, which other animals have you seen in or around your home? ☐ Cats ☐ Dogs ☐ Rats ☐ Mice ☐ Bats ☐ Cockroaches ☐ Other ________________

Kung Meron, anong mga hayop ang makikita sa paligid ng iyong bahay?

☐ Pusa ☐ Aso ☐ Daga ☐ Bubwit ☐ Paniki ☐ Ipis ☐ At iba pa:______________

25. Did you attend any social gatherings within the residential complex in the last two months? ☐ Yes ☐ No

Ikaw ba ay dumalo o nakadalo sa anumang social na pagtitipon sa loob ng residential complex nyo sa huling dalawang buwan? ☐ Oo ☐ Hindi

25.1.If yes, what was the gathering for (e.g., EID)(add some answers plus an other with open ended)

Kung Oo, ano or para saan ang pagtitipon na iyon? (magdagdag ng ilang mga sagot kasama ang isa pa or mga event na di pa natapos)

Gathering 1: (description) ______________________ Number of women attending (estimate): _______

Pagtitipon 1: (isalarawan) __________________ Bilang ng mga kababaihan na dumalo o dadalo_____

Gathering 2: (description) ______________________ Number of women attending (estimate): _______

Pagtitipon 2: (isalarawan) __________________ Bilang ng mga kababaihan na dumalo o dadalo_____

26. Do you socialize with the women in your villa? ☐ Yes ☐ No

Ikaw ba ay nakikisalamuha sa mga babae sa iyong villa? ☐ Oo ☐ Hindi

26.1.If yes, what socialization do you do?

☐Watch TV/movies/you tube together on a shared laptop/computer ☐play volleyball

☐Attend parties ☐share meals ☐sing songs

☐Other _________________________

Kung Oo, anong klase ng kaganapan or pagtitipon ang ginagawa mo?

☐ Magkasama sa panonood ng TV / pelikula / Youtube sa isang laptop / computer

☐Dumalo sa mga party ☐Magkasalo sa pagkain ☐Kumanta /Kantahan

☐ At iba pa:

27. Did you attend any social gathering last month? ☐ Yes ☐ No

Dumalo ka ba ng anumang mga pagtitipon noong nakaraang buwan? ☐ Oo ☐ Hind

27.1 If yes when? _________________________ Kung Oo, kalian? _________________________

27.2 What was the nature of the social gathering? _________________________

Ano ang klase ng pagtitipon? _________________________

CONTACTS

28. Have you had any contact with a known or suspected MERS-CoV patient? ☐ Yes ☐ No

Ikaw ba ay nagkaroon ng anumang contact sa isang kilala o pinaghihinalaang mga pasyente MERS-CoV? ☐ Oo ☐ Hindi

28.1 If yes, who did you come in contact with? (name)________________________

Kung Oo, sino ito? (ibigay ang pangalan) ______________________________

28.2 yes, what was the nature of the contact (choose all that apply)

☐Shared a bedroom ☐shared a kitchen ☐shared a bathroom ☐shared a meal

☐shared transportation to or from work

☐had direct contact with patient (e.g., hugged, touched patient)

☐had no direct contact but spoke to patient (within 3 feet)….

☐ Other_____________________

Kung Oo, ano o paano kayo nagkaroon ng contact (piliin ang lahat na naaangkop)

☐ Magkasama or share ng isang kwarto ☐ Magkashare ng kusina

☐ Magkashare ng banyo ☐ Magkashare ng pagkain

☐ Magkashare ng sasakyan papunta at pauwi ng trabaho

☐ nagkaroon ng direktang kontak sa mga pasyente (eg, niyakap, hinawakan pasyente)

☐walang direktang kontact ngunit nakipag ugap sa pasyente (sa loob ng 3 talampakan)

☐ At iba pa ____________

29 Have you had contact with a roommate or housemate with respiratory, gastrointestinal symptoms or fever in the last 4 weeks? ☐ Yes ☐ No

29.1 If yes, who did you have contact with? (list)

Contact 1:_____________________________

Contact 2:_______________________________

Contact 3:_______________________________

Contact 4:_______________________________

(add more if necessary)

Nagkaroon ng kontak sa isang kasama sa kuwarto o kasambahay na may sakit sa paghinga, Gastrointestinal sintomas o lagnat sa huling 4 na linggo? ☐ Oo ☐ Hindi

Kung Oo, sino or kani-kanino ka nakipag-ugnayan? (listahan)

Contact 1:_____________________________

Contact 2:_______________________________

Contact 3:_______________________________

Contact 4:_______________________________

(add more if necessary)

OCCUPATIONAL EXPOSURES

30 Where do you work? ____________________________

Saan ka nagtatrabaho? __________________________

31 What building are you working in? ____________________________
Ano ang pangalan ng gusali o lokasyon na pinagtatrabahuhan mo?

____

32 If working in Princess Nora University, do you have contact with any students or faculty? ☐ Yes ☐ No
Kung ikaw ay nagtatrabaho sa Princess Nora University, Mayroon ba kayong ka-ugnayan sa anumang

mga mag-aaral o mga kasapi na guro? ☐Oo ☐ hindi

32.1 If yes, were any of these students/faculty members sick in the last two weeks? ☐ Yes ☐ No
Kung Oo, meron ba sa alinman nitong mga mag-aaral o kasaping mga guro ay may sakit sa huling

dalawang linggo? ☐Oo ☐ hindi

33 Where is your current primary employment? ___________________

33.1 How long have you worked at this location? ___ ___ Years___ ___ Months

33.2 What is the address of your work? ______________________

Saan ang iyong kasalukuyang pangunahing trabaho? ______________

Gaano ka na katagal nagtrabaho sa lokasyon na ito? _______ taon______buwan

Ano ang address ng iyong trabaho? _____________________

34 What is/are your job/jobs at this location? (tick all that apply)

☐ Technician ☐ Cleaning ☐ Engineer ☐ Other _____________________

34.1 Of the listed options, which you selected, which is your primary job?

____

Ano ang / iyong mga trabaho / sa lokasyon na ito? (lagyan ng tsek ang lahat ng naaangkop)

☐Tekniko ☐ Paglilinis ☐Engineer ☐ At iba pa ___________________

Sa mga nakalista pagpipilian, na kung saan na iyong pinili, ano sa mga ito ang iyong pangunahing trabaho? ___________________

35 How often in the week do you work at this location?

Day Working? Hour start Hour end

Monday ☐ YES __________ __________

Tuesday ☐ YES __________ __________

Wednesday ☐ YES __________ __________

Thursday ☐ YES __________ __________

Friday ☐ YES __________ __________

Saturday ☐ YES __________ __________

Sunday ☐ YES __________ __________

Gaano kadalas sa isang linggo ka nagtatrabaho sa lokasyon na ito?

Araw Nagtatrabaho? Umpisa ng trabaho Tapos ng Trabaho

Lunes ☐ Oo __________ __________

Martes ☐ Oo __________ __________

Miyerkules ☐ Oo __________ __________

Huwebes ☐ Oo __________ __________

Biyernes ☐ Oo __________ __________

Sabado ☐ Oo __________ __________

Linggo ☐ Oo __________ __________

36 If you hold only one job, what do you do in the evenings after 14:00 or 15:00 until you go to sleep?____

Kung ikaw ay mayroon lamang isang trabaho, ano ang ginagawa mo sa gabi sa pagitan ng 14:00 o 15:00? ____

37 **How frequent do you go for shopping?**

____

Gaano ka kadalas pumunta para sa pamimili?**__________________________**

37.1 When was the last time (date) that you went for shopping?______________________

Kailan (petsa) ka huling nagpunta para sa pamimili?**______________________**

37.2 Where did you do your last shopping?_______________________

Saan ka huling namili?**________________________**

38 Do you usually play volleyball with other sisters in the compound?_________________________

Ikaw ba ay karaniwang naglalaro ng volleyball kasama ng iba pang mga babae sa compound?

____

38.1 When was the last time you played volleyball with other sisters in the compound?

____

Kailan ka huling naglaro ng volleyball kasama ng iba pang mga babae sa compound?

____

39 Do you usually watch TV with roommates/ housemates in the compound?

____

Ikaw ba ay karaniwang nanonood ng TV kasama ng iyong mga roommate / kasambahay sa compound? **____**

39.1 If yes, when was the last time you watched TV with roommates/ housemates in the compound?**____**

Kung Oo, kalian ang huling panahon na nanood ka ng TV kasama ng iyong mga roommate / kasambahay sa compound? **____**

40 What personal protective equipment do you usually wear when working at your primary job?

☐ No protective equipment used ☐ Gloves ☐ Coveralls
☐ Dust masks ☐ Boots or boot covers ☐ Respirators
☐ Eye protection (goggles, safety glasses) ☐ Others:__________________________

Ano ang mga personal na proteksiyon kagamitan at karaniwang iyong isinusuot kapag nagtatrabaho sa iyong pangunahing trabaho?

☐ Walang kagamitang pangprotekcyonna ginamit ☐ Guwantes
☐ Coveralls ☐ Dust masks ☐ Bota ☐ Respirators
☐ Proteksyon sa mata (goggles) ☐ At Iba pa: ___________________________

41 How often do you usually wash your hands while working at your primary job (check all) ☐ At mealtimes ☐ Before and after each animal related task

☐ At bathroom times ☐ The beginning and end of the day ☐ Rarely

Gaano kadalas ka kadalasang naghuhugas ng inyong mga kamay habang nagtatrabaho sa iyong pangunahing trabaho (i-check ang lahat)

☐ sa oras ng kainan ☐ Bago at pagkatapos ng bawat gawain na may kaugnayan sa hayop

☐ sa oras ng pagba banyo ☐ Sa pag simula at pagtatapos ng araw ☐ Madalang

ANIMAL EXPOSURES IN/AROUND THE HOME where you live

42. Were any animals (e.g. camels, sheep, goats, cattle, horses, cats, dogs, birds) kept in or around your home in the last six months? ☐ YES ☐ NO ☐ UNKNOWN

Mayroon bang mga hayop (eg kamelyo, tupa, kambing, baka, kabayo, pusa, aso, ibon) sa inyong paligid ng iyong tahanan sa huling anim na buwan? ☐ Meron ☐ Wala ☐ Di Alam

42.2 Which animals?

☐ kamelyo ☐ tupa ☐ kambing ☐ baka ☐ kabayo ☐ pusa ☐ aso, ☐ bon

43. In the last six months, did you have any contact with any carcasses, body fluids, secretions, urine or excrement of camels in or around your home? ☐ YES ☐ NO ☐ UNKNOWN

Sa huling anim na buwan, ikaw ba ay mayroon anumang contact sa mga bangkay, likido sa katawan, secretions, ihi o dumi ng kamelyo sa o sa paligid ng iyong bahay? ☐ Meron ☐ Wala ☐ Di Alam

44. In the last six months, did you have any contact with any camel bedding, stray of feed in or around your home? ☐ YES ☐ NO ☐ UNKNOWN

Sa huling anim na buwan, ikaw ba ay mayroon anumang contact sa mga gamit ng kamelyo (tulugan), o nagkalat/nagbigay ng kanilang mga pagkain sa paligid ng iyong bahay? ☐ Oo ☐ Hindi ☐ Di Alam

45. Do others living in your household (e.g., domestic help or relative) frequently visit or work on a farm or market where camels are kept or sold? ☐ YES ☐ NO ☐ UNKNOWN

Mayroon ba sa ibang nakatira sa inyong sambayan (eg, domestic helper/ka tulong o kamag-anak) ang madalas bumisita o nagtatrabaho sa isang sakahan o pamilihan kung saan nagbebenta ng kamelyo? ☐ Meron ☐ Wala ☐ Di Alam

46. Have others living in your household (e.g., domestic help or relative) had visited or worked in the in the past 2 weeks at a farm or market where camels are kept or sold? ☐ YES ☐ NO ☐ UNKNOWN

Sa ibang nakatira sa inyong sambahayan (eg, domestic helper/ka tulong o kamag-anak) bumisita ba sila o nagtrabaho sa isang sakahan o pamilihan ng kamelyo sa huling dalawang (2) lingo? ☐ Oo ☐ Hindi ☐ Di Alam

47. **Have others living in your household (e.g., domestic help or relative) had direct contact with camels in the past 2 weeks?** ☐ YES ☐ NO ☐ UNKNOWN

    Sa ibang nakatira sa inyong sambahayan (eg, domestic helper/ka tulong o kamag-anak) nagkaroon ba ng direktang kontak sa mga kamelyo sa nakaraan 2 linggo? ☐ Oo ☐ Hindi ☐ Di Alam

FOOD EXPOSURES

The following series of questions are focused on food exposures in the last month

Ang mga sumusunod na serye ng mga katanungan ay nakatutok sa mga exposure ng pagkain sa nakaraang buwan

During the past six months, how often on average did you consume any of the following products:

Sa panahon ng nakaraang anim na buwan, gaano kadalas sa average na ubusin mo ang alinman sa mga sumusunod na produkto

48. **Did you drink unpasteurized camel milk? ☐ YES ☐ NO ☐ UNKNOWN**

    Uminom ka ba unpasteurized kamelyo gatas? ☐ Oo ☐ Hindi ☐ Di Alam

49. **Did you use camel urine, for example, for medicinal purposes? ☐YES ☐ NO ☐ UNKNOWN**

Sa layunin ng panggagamot, gumamit ka ba ng ihi ng kamelyo? ☐ Oo ☐ Hindi ☐ Di Alam

50. **Did you drink camel urine? ☐YES ☐ NO ☐ UNKNOWN**

    Uminom ka ba ng ihin ng kamelyo? ☐ Oo ☐ Hindi ☐ Di Alam

TRAVEL HISTORY AND EXPOSURES

51. **During the last 2 months have you travelled outside KSA? ☐ YES ☐ NO ☐ UNKNOWN**

Sa panahon ng huling 2 buwan ikaw ba ay nakapaglakbay sa labas KSA? ☐ Oo ☐ Hindi ☐ Di Alam

51.1 If yes, what countries/regions have you visited?

Country Region/City Approximate Dates

_________________________ ___________________________ __________________________
_________________________ ___________________________ __________________________
_________________________ ___________________________ __________________________
_________________________ ___________________________ __________________________
_________________________ ___________________________ __________________________

Kung oo, ano mga bansa / rehiyon ang iyong mong binisita?

Bansa Rehiyon/Syudad Petsa

_________________________ ___________________________ __________________________
_________________________ ___________________________ __________________________
_________________________ ___________________________ __________________________
_________________________ ___________________________ __________________________

_________________________ ___________________________ __________________________

52. **While traveling, have you attended any mass gatherings (e.g., weddings, festivals or religious pilgrimages) outside KSA where there were large numbers of people together?** ☐ YES ☐ NO ☐ UNKNOWN

52.1 If yes, specify event(s) and location:

____

Sa pahanon ng iyong naglalakbay, pumasok o dumalo kaba sa anumang malakihang pagtitipon (halimbawa, weddings, festival o pilgrimages relihiyon) sa labas ng KSA kung saan mayroong malaking bilang ng mga tao na magkasama? ☐ Oo ☐ Hindi ☐ Di Alam

Kung Oo, tukuyin ang (mga) kaganapan at lokasyon:

____

53. **When you travelled, did you have direct or indirect contact with dromedary camels while outside of KSA?** ☐ YES ☐ NO ☐ UNKNOWN

54. **In the last month, have you visit any health care facility outside of KSA? ☐ YES ☐ NO ☐ UNKNOWN**

54.1 If yes, where (city, country, hospital name) ______________

Sa mga nakaraang buwan, ikaw ba ay bumisita sa anumang health care facility sa labas ng KSA?
☐ Oo ☐ Hindi ☐ Di Alam
Kung oo, saan (Syudad, Bansa, pangalan ng ospital) ____________________

SIGNS AND SYMPTOMS

Palatandaan at Sintomas

55. Are you sick today with fever and/or cough? ☐ YES ☐ NO

a. If yes, when did your symptoms start (DD/MM/YYYY): ____/___/______

Ikaw ay may sakit ngayon at may lagnat at / o pag-ubo? ☐ Oo ☐ Hindi
Kung Oo, kailan nagsimula ang iyong mga sintomas
(petsa: araw/buwan/taon):____/____/___

56. Did you experience any respiratory signs or symptoms during the last four weeks?

☐ YES ☐ NO ☐ UNKNOWN

If yes, when did these symptoms start (DD/MM/YYYY): ____/___/______

Nakaranas ka ba ng anumang respiratory (sakit sa paghinga) na palatandaan o sintomas

sa loob ng huling apat na linggo? ☐ Oo ☐ Hindi ☐ Di Alam

Kung Oo, kailan nagsimula ang mga sintomas na ito (petsa:
araw/buwan/taon):____/____/____

57. If you answered yes to either #1 or #2, please indicate which symptoms:

Symptom Today Last 4 weeks

Dry Cough ☐ YES ☐ NO ☐ UNKNOWN ☐ YES ☐ NO ☐ UNKNOWN

Productive Cough ☐ YES ☐ NO ☐ UNKNOWN ☐ YES ☐ NO ☐ UNKNOWN

Phlegm ☐ YES ☐ NO ☐ UNKNOWN ☐ YES ☐ NO ☐ UNKNOWN

Runny nose ☐ YES ☐ NO ☐ UNKNOWN ☐ YES ☐ NO ☐ UNKNOWN

Sore throat ☐ YES ☐ NO ☐ UNKNOWN ☐ YES ☐ NO ☐ UNKNOWN

Fever ☐ YES ☐ NO ☐ UNKNOWN ☐ YES ☐ NO ☐ UNKNOWN

Shortness of breath ☐ YES ☐ NO ☐ UNKNOWN ☐ YES ☐ NO ☐ UNKNOWN

Muscle pain ☐ YES ☐ NO ☐ UNKNOWN ☐ YES ☐ NO ☐ UNKNOWN

Diarrhea ☐ YES ☐ NO ☐ UNKNOWN ☐ YES ☐ NO ☐ UNKNOWN

Chest Pain ☐ YES ☐ NO ☐ UNKNOWN ☐ YES ☐ NO ☐ UNKNOWN

Vomiting ☐ YES ☐ NO ☐ UNKNOWN ☐ YES ☐ NO ☐ UNKNOWN

Rashes ☐ YES ☐ NO ☐ UNKNOWN ☐ YES ☐ NO ☐ UNKNOWN

Kung sumagot ka ng Oo sa alinman sa # 1 o # 2, mangyaring ipahiwatig kung aling mga sintomas:

Sintomas Kasalukuyang Araw (Today) Huling 4 na Linggo (Last 4 weeks)

Tuyong Ubo ☐ OO ☐ HINDI☐ DI MATUKOY ☐ OO ☐ HINDI☐ DI MATUKOY

Produktibong ubo ☐ OO ☐ HINDI☐ DI MATUKOY ☐ OO ☐ HINDI☐ DI MATUKOY

Plema ☐ OO☐ HINDI☐ DI MATUKOY ☐ OO ☐ HINDI☐ DI MATUKOY

Sinisipon ☐ OO☐ HINDI☐ DI MATUKOY ☐ OO ☐ HINDI☐ DI MATUKOY

Namamagang lalamunan ☐ OO☐ HINDI☐ DI MATUKOY ☐ OO ☐ HINDI☐ DI MATUKOY

Lagnat ☐ OO ☐ HINDI☐ DI MATUKOY ☐ OO ☐ HINDI☐ DI MATUKOY

Pangangapos ng hininga ☐ OO ☐ HINDI☐ DI MATUKOY ☐ OO ☐ HINDI☐ DI MATUKOY

Pananakit ng kalamnan ☐ OO ☐ HINDI☐ DI MATUKOY ☐ OO ☐ HINDI☐ DI MATUKOY

Pagtatae ☐ OO ☐ HINDI☐ DI MATUKOY ☐ OO ☐ HINDI☐ DI MATUKOY

Pananakit ng dibdib ☐ OO ☐ HINDI☐ DI MATUKOY ☐ OO ☐ HINDI☐ DI MATUKOY

Pagsusuka ☐ OO ☐ HINDI☐ DI MATUKOY ☐ OO ☐ HINDI☐ DI MATUKOY

Pamamantal ☐ OO ☐ HINDI☐ DI MATUKOY ☐ OO ☐ HINDI☐ DI MATUKOY

58. Did you seek medical care? ☐ YES ☐ NO ☐ UNKNOWN

If yes, where did you seek medical care (name and address of medical facility)? ____

If yes, when did you seek medical care (DD/MM/YYYY): ____/___/______

Ikaw ba ang kumonsulta sa manggagamot? ☐ OO ☐ HINDI☐ DI MATUKOY

Kung Oo, saang ospital ka nagpakonsulta? (pangalan at address ng mga medikal na pasilidad)?

____

Kung Oo, kailan ka nagpakonsulta? (petsa: araw/buwan/taon): ____/___/______

59. Where you hospitalized during the course of your illness? ☐ YES ☐ NO ☐ UNKNOWN

59.1 If yes, when were you hospitalized (DD/MM/YYYY): ____/___/______

59.2 If yes, which hospital did you receive treatment(s)? (name and address)

____

Ikaw pa ay na-ospital sa mga panahon ng iyong pagkaskasakit? ☐ OO ☐ HINDI☐ DI MATUKOY

Kung Oo, kelan ka na-ospital (petsa: araw/buwan/taon): ____/___/______

Kung Oo, saan o anong ospital ka nakatanggap ng (mga) paggamot? (pangalan at address ng ospital) ____

MEDICAL HISTORY AND RELATED EXPOSURES

KASAYSAYAN MEDIKAL AT MGA KAUGNAY NA PAGKAKALANTAD

60. Do you currently smoke tobacco (ex. cigarettes, cigars, shisha)?
☐ Daily ☐ Less than daily ☐ Not at all ☐ Unknown
Ikaw ba sa kasalukuyan ay nagsisigarilyo (nagtatabako)? (ex. Sigarilyo, tabako, shisha)?
☐ Araw-araw ☐ Madalang sa araw-araw ☐ Hindi ☐ Di Matukoy

61. Do you share the same cigarette, cigar, shisha? ☐ YES ☐ NO ☐ UNKNOWN
Ikaw ba ay nakikibahagi ng parehong sigarilyo, cigar, shisha? ☐ OO ☐ HINDI☐ DI MATUKOY

62. Have you smoked tobacco daily in the past? ☐ YES ☐ NO ☐ UNKNOWN

Ikaw ba ay nagsisigarilyo ng tabako araw-araw sa mga panahong nakalipas ? ☐ OO ☐ HINDI ☐ DI MATUKOY

63. Is there any hereditary disease running in your family? ☐ YES ☐ NO ☐ UNKNOWN

63.1 If yes, please specify the disease(s): _______________________________________

Mayroon ba sa iyong pamilya ang anumang mga sakit na namamana? ☐ OO ☐ HINDI ☐ DI MATUKOY

Kung Oo, mangyaring tukuyin ang (mga) sakit: ________________________________

64. Do you currently have any chronic illness (ex. asthma, cancer, diabetes)? ☐ YES ☐ NO ☐ UNKNOWN

64.1 If yes, please specify the disease(s): ___________________________________

Sa kasalukuyan mayroon ka bang anumang mga hindi gumagaling na sakit (ex. Hika, kanser, diabetes)? ☐ OO ☐ HINDI☐ DI MATUKOY

Kung Oo, mangyaring tukuyin ang (mga) sakit: _________________________________

65. **Have you taken medications regularly in the last six months? ☐ YES ☐ NO ☐ UNKNOWN**

65.1 If yes, what medications do you regularly take? (list all)

____

____

Ikaw ba ay may mga gamot na regular na iniinom sa huling anim na buwan? ☐ OO ☐ HINDI ☐ DI MATUKOY

Kung Oo, anong gamot ang regular mong iniinom? (ilista ang lahat)

____

____

66. **Have you taken any traditional medications in the last six months? ☐ YES ☐ NO ☐ UNKNOWN**

If yes, which traditional medications (list all)

____

____

Ikaw ba ay may iniinom na anumang tradisyonal na mga gamot sa huling anim na buwan? ☐ OO ☐ HINDI ☐ DI MATUKOY

Kung Oo, anong tradisyunal na gamot? (ilista ang lahat)

____

____

67. **What is your height _______ cm** Ano ang iyong taas ________ cm

68. **What is your weight ________.____ kg** Ano ang iyong timbang ________._____kg

69. **How many bars of soap to you use per month? _____**

Gaano karaming mga bar ng sabon ang nagagamit mo sa bawat buwan? ________

70. **How frequently do you bathe? ___________ per day/per week**

Gaano ka kadalas maligo? ____________ beses isang araw/ isang lingo

71. **What is the highest level of education? ☐ Primary school ☐ High School ☐ University ☐ Post Graduate degree**

Ano ang pinakamataas na antas ng iyong edukasyon? ☐ Mababang Paaralan ☐ Mataas na paaralan ☐ Unibersidad ☐ Post Graduate degree

72. **Have you visited anyone in the hospital in the last 2 months?** ☐ YES ☐ NO ☐ UNKNOWN

If yes, was the person sick with respiratory illness (cough, breathing problems)? ☐ YES ☐ NO ☐ UNKNOWN

72.1 If yes, at what hospital (regions, city, district)______________________________

72.2 If yes, what was your relationship to the person in the hospital? ☐ Close family ☐ Extended family ☐ Friend ☐ Other____________

Ikaw ba ay mayroong sinumang binisita sa ospital sa huling 2 buwan? ☐ OO ☐ HINDI☐ DI MATUKOY

Kung Oo, ang taong ito ba ay may sakit paghinga/respiratory (tulad ng ubo, at iba pang mga problema sa paghinga)? ☐ OO ☐ HINDI☐ DI MATUKOY

Kung Oo, saan o anong ospital (mga rehiyon, lungsod, distrito) ____________________

Kung oo, ano ang iyong relasyon sa taong nasa ospital? ☐ Malapit na kamag-anak ☐ Extended family ☐ Kaibigan ☐ At iba pa____________

73. **Had you heard of MERS Coronavirus before this outbreak?** ☐ YES ☐ NO

73.1 If yes, what was the source of your information? ☐ Ministry of Health ☐ TV ☐ Supervisor ☐ Other ___________________

Mayroon ka bang napapakinggan tungkol sa MERS Coronavirus noon pa man bago pa ito naging outbreak? ☐ OO ☐ HINDI

Kung Oo, saan o ano ang pinagmulan ng iyong impormasyon? ☐ Ministry of Health ☐ TV ☐ Supervisor ☐ Other ________________

74. In the last month, how many times have you been visited by a health care professional about MERS-CoV?____

74.1 How many times have samples been collected from you? _________

74.2 What samples were collected? ☐ NP ☐OP ☐Blood ☐other___________

74.3 What dates were samples collected from you?

Sa nakaraang buwan, ilang beses ka na binisita ng isang propesyonal ng pangkalusugang pag-aalaga tungkol sa MERS-CoV? ______________

Ilang beses na samples na nakolekta mula sa iyo? ____________________

Anong sample ang nakolekta sa iyo? ☐ NP ☐OP ☐Blood ☐At iba pa___________

Anong petsa ang sample na nakolekta mula sa iyo? ___________________

75 May we contact you again with follow up questions or clarifications? ☐ YES ☐ NO

Telephone number of subject: ____

Maari ba kaming makipag-ugnayan muli sa iyo para sa susunod pang mga katanungan o paglilinaw? ☐ OO ☐ HINDI Numero ng telepono: _________________________

The following questionnaire was administered individually to participants by a trained interviewer from the Ministry of Health or Institut Pasteur. The interviewer read each question aloud and recorded the participants answers directly into the questionnaire.

Outbreak Investigation Questionnaire

GENERAL INFORMATION

PANGKALAHATANG IMPORMASYON

1. Subject ID: ___ ___ ___ ___ ___ ___ ___ ___

Numero ng ID: ___ ___ ___ ___ ___ ___ ___ ___

2. Subject Name: First name ________________ Surname_________________

Panglan: ___________________________ Apelyido: _______________

SECONDARY JOB

3. Do you hold other jobs aside from your primary job? ☐ YES ☐ NO

3.1. If yes, what is/are your other job(s)? ___________________________________

3.2. If yes, where is this other job? ______________________________

3.3. If yes, how often in the week do you work at this second location?

4. Day Working? Hour start Hour end

Monday ☐ YES __________ __________

Tuesday ☐ YES __________ __________

Wednesday ☐ YES __________ __________

Thursday ☐ YES __________ __________

Friday ☐ YES __________ __________

Saturday ☐ YES __________ __________

Sunday ☐ YES __________ __________

Ikaw ba ay may iba pang trabaho sa ibang lugar maliban sa lokasyon na ito? ☐ Meron ☐ Wala

Kung oo, ano ang / iyong (mga) iba pang mga trabaho?_________________________

Kung oo, gaano kadalas sa isang linggo ka nagtatrabaho dito sa pangalawang lokasyon?

Araw Nagtatrabaho? Umpisa ng trabaho Tapos ng Trabaho

Lunes ☐ Oo __________ __________

Martes ☐ Oo __________ __________

Miyerkules ☐ Oo __________ __________

Huwebes ☐ Oo __________ __________

Biyernes ☐ Oo __________ __________

Sabado ☐ Oo __________ __________

Linggo ☐ Oo __________ __________

5. If the second location is a health care facility:

5.1. What is the name of the health care facility in which you work? _________________

5.2. What is the location of the health care facility in which you work? ________________

5.3. Where in the health care facility do you work? _________________________________

5.4. What/which department(s) in this health care facility do you work? ________________________

5.5. Do you have any contact with biological specimens during your work? ☐ Yes ☐ No ☐Unknown

5.6. Do you handle soiled patient linens during your work? ☐ Yes ☐ No ☐Unknown

5.7. Have you worked in a room where there was a MERS-CoV patients? ☐ Yes ☐ No ☐Unknown

Kung ang pangalawang lokasyon/trabaho ay isang pasilidad ng pangangalagang pangkalusugan:

Ano ang pangalan ng pangangalagang pangkalusugan (health care facility) kung saan ka nagtatrabaho: ____

Ano ang lokasyon ng pangkalusugang pag-aalaga pasilidad kung saan ka nagtatrabaho ____

Saan sa mga pasilidad ng pangangalagang pangkalusugan ka nagtatrabaho?

____

Ano, o sa aling (mga) departamento ng pasilidad na ito sa pangangalaga ng kalusugan ka nagta trabaho?__________________________

Mayroon ka bang anumang mga contact sa mga biological ispesimen sa panahon ng iyong trabaho? __________________________ ☐ Meron ☐ Wala ☐ Di Alam

Humahawak ka ba ng maruming gamit ng pasyente tulad ng linens sa panahon ng iyong trabaho? ☐ Oo ☐ Hindi ☐ Di Alam

Ikaw ba ay nakapagtrabaho sa isang silid kung saan nagkaroon ng mga pasyenteng may MERS-CoV? ☐ Oo ☐ Hindi ☐ Di Alam

6. In the last 6 weeks, have you worked in a health care facility? ☐ Yes ☐ No

6.1. What is the name of the health care facility in which you worked? __________________

6.2. What is the location of the health care facility in which you worked? ____________________

6.3. Where in the health care facility did you work? _______________________________

6.4. What/which department(s) in this health care facility did you work?______________________________

6.5. Did you have any contact with biological specimens during your work? ☐ Yes ☐ No ☐Unknown

6.6. Did you handle soiled patient linens during your work? ☐ Yes ☐ No ☐Unknown

6.7. Did you work in a room where there was a MERS-CoV patients? ☐ Yes ☐ No ☐Unknown

Sa huling 6 na linggo, ikaw aba y nagtrabaho sa isang pasilidad ng pangangalaga ng kalusugan? ☐ Oo ☐ Hindi

Ano ang pangalan ng mga pasilidad ng pangangalagang pangkalusugan sa kung saan ka nagtrabaho? ____________________________

Ano ang lokasyon ng mga pasilidad ng pangangalagang pangkalusugan sa kung saan ka nagtrabaho?________________________

Saan sa mga pasilidad ng pangangalagang pangkalusugan ka nagtatrabaho?________________

Ano, o sa aling (mga) departamento ng pasilidad na ito sa pangangalaga ng kalusugan ka nagta trabaho?__________________________

Mayroon ka bang anumang mga contact sa mga biological ispesimen sa panahon ng iyong trabaho? __________________________ ☐ Meron ☐ Wala ☐ Di Alam

Humahawak ka ba ng maruming gamit ng pasyente tulad ng linens sa panahon ng iyong trabaho? ☐ Oo ☐ Hindi ☐ Di Alam

Nagtatrabaho ka ba sa isang silid kung saan nagkaroon ng mga pasyente MERS-CoV?

PERSONAL PROTECTIVE EQUIPMENT AND HYGIENE PRACTICES if you also worked in a health care facility in the last 3 months

7. What personal protective equipment do you usually wear when working at the health care facility?

☐ No protective equipment used ☐ Gloves ☐ Coveralls

☐ Dust masks ☐ Boots or boot covers ☐ Respirators ☐ Eye protection (goggles, safety glasses) ☐ Others:____________________________

PERSONAL NA KAGAMITANG PANGHARANG AT KALINISAN NA NAKASANAYAN kung ikaw rin ay nagtrabaho sa isang pasilidad ng pangangalagang pangkalusugan sa nakaraang 3 buwan

Ano ang personal na proteksiyon kagamitan ay karaniwang mo magsuot kapag nagtatrabaho sa mga pasilidad ng pangangalaga ng kalusugan?

☐ Walang kagamitang pangproteksyon na ginamit ☐ Guwantes ☐ Coveralls

☐ Dust masks ☐ Boots or boot covers ☐ Respirators

☐ Proteksyon sa mata (goggles) ☐ At Iba pa: ____________________________

8. **How often do you usually wash your hands while working at the health care facility (check all)**

☐ At mealtimes ☐ Before and after each animal related task ☐ At bathroom times

☐ The beginning and end of the day ☐ Rarely

Gaano kadalas ka kadalasang naghuhugas ng inyong mga kamay habang nagtatrabaho sa iyong pangunahing trabaho (i-check ang lahat)

☐ sa oras ng kainan ☐ Bago at pagkatapos ng bawat gawain na may kaugnayan sa hayop
☐ sa oras ng pagba banyo ☐ Sa pag simula at pagtatapos ng araw ☐ Madalang

Pregnancy

3. Are you pregnant? ☐ YES ☐ NO ☐ UNKNOWN

Ikaw ba ay buntis? ☐ OO ☐ HINDI☐ DI MATUKOY

4. If no, were you pregnant in the last six months? ☐ YES ☐ NO ☐ UNKNOWN

Kung Hindi, ikaw ba ay buntis sa huling anim na buwan? ☐ OO ☐ HINDI☐ DI MATUKOY
